# Disinformation and calculated care beyond the Global North: comparing refugee discourses in Australia and India

**DOI:** 10.3389/fsoc.2024.1358556

**Published:** 2024-12-11

**Authors:** Sukhmani Khorana, Nisha Thapliyal

**Affiliations:** ^1^School of the Arts and Media, University of New South Wales Sydney, Kensington, NSW, Australia; ^2^School of Education, The University of Newcastle, Callaghan, NSW, Australia

**Keywords:** disinformation, care, refugees, Muslim, Rohingya, Biloela, India, Australia

## Abstract

This article explores what “care” looks like in the specific context of Muslim refugees and asylum seekers within the dominant discourse of humanitarianism. India and Australia are chosen for this comparative analysis because our aim is to emphasise multidimensional anti-Muslim alliances that are now in place in both contexts between the governments and official and unofficial media that influence humanitarian policies and practice. We argue that the “information disorder” that dominates current media ecologies about Muslim refugees in both countries is produced at this nexus of official agents—both state and media institutions—as well as social media content produced by local and global actors that perpetuate anti-Muslim bias. More specifically, this article examines how India has responded to emergencies involving the Rohingya refugees, and Australia’s treatment of post-9/11 Muslim refugees and asylum seekers. We demonstrate that these states and the media they sponsor are linked to the use of disinformation, or deliberately inaccurate information to seed and perpetuate Islamophobic sentiments and thereby practice a form of “calculated care”. The examples in this article highlight the need to build on our understanding of what constitutes humanitarian care towards vulnerable and stateless populations. Furthermore, they call for response strategies that take into cognizance the fact that Islamophobia has been institutionalized in the public sphere in order to promote culturally supremacist discourses of traditional values as well as national security.

## Introduction

### Emotions and care in the context of migration

The introductory section of this article is organised in two parts to introduce (a) why emotions and the ethics of care matter in the context of global migration; and (b) what is “information disorder” and how disinformation about migration is a particularly pertinent phenomenon to examine in terms of how it mobilizes public emotions using a range of mainstream, state-sponsored, and social media.

In migration research as well as popular understandings of migration, the emotions of those who migrate are considered secondary to how the receiving societies feel about, and adjust to their arrival (Khorana, 2023). These so-called “public attitudes” to migration have an enormous impact on political promises during economic crises, and hence on long-term migration policy. In recent times, these shifts were noticed during international border closures across many Global North and Global South nations during the COVID-19 pandemic. The closures were, in many cases, accompanied by misinformation and disinformation about migrants and the virus on mainstream and social media platforms, leading to emotive public and political responses. Examples include the fear induced in those of “East Asian” appearance who were subjected to increased racism in the wake of the pandemic in countries such as the US and Australia (Hahm et al., 2021; Reny and Barreto, 2020), and the precarity and uncertainty faced by international students and temporary migrants as a result of not being eligible for welfare payments (Gomes et al., 2021).

Empirical research has also now been published about COVID-related sentiments in relation to migration that is specific to harms and/or acts of care circulating in the mediated sphere. Notable examples include Banaji and Bhat’s exhaustive study of the Islamophobic media landscape which enabled Muslim pilgrims belonging to the Tablighi Jamat congregation to be blamed for the pandemic (Banaji and Bhat, 2019, 2020); Croucher et al.’s study of how social media use increases the likelihood of someone developing and expressing anti-Asian sentiments (Croucher et al., 2020), Ziems et al.’s research on the spread of anti-Asian hate speech through Twitter (Ziems et al., 2020), and von Ana Makhashvili’s use of the term “the affective economy of anxiety” to describe how the far-right is using social media to mobilise in Germany and elsewhere in the wake of the pandemic (Makhashvili and Medeiros, 2020). While the present article is not focused on COVID and migration itself, this recent crisis highlights the importance of an emotions and affect lens for examining migration itself as well as looking closely at how the circulation of large-scale inaccurate and decontextualised information about migrants can cause serious harm.

### What humanitarian treatment of asylum seekers entails

According to the United Nations High Commissioner for Refugees (UNHCR), “Seeking asylum is a human right and every person in the world has the right to apply for asylum if they are fleeing conflict or persecution”. Further, these asylum seekers must not be expelled or returned to situations of danger – this is also known as the principle of non-refoulement and is enshrined in the 1951 Refugee Convention. The UNHCR expects all countries to adhere to this principle as it is part of human rights law and customary international law.

Despite this, policy and practice within nations and across time have varied a great deal. In the Australian case, for instance, mandatory offshore detention of all arrivals by sea has been in place since 2012 (Refugee Council of Australia, 2020) in spite of legal challenges and the UNHCR’s explicit advocacy against detention and for protection of asylum seekers at sea. The regime of offshore mandatory detention has also become increasingly more militarized and securitised with the introduction of “border protection” policies in recent years (Laney et al., 2016: p. 136). The Pacific islands of Manus and Nauru were a focal point of these policies and associated detention centres; they were in operation despite repeated criticism from international human rights organizations. Such “cruelty” has persisted despite the outward appearance of Australia committing to the UN Refugee Convention and humane approaches to resettling refugees whose asylum claims have been found to be legitimate. Grewcock notes that despite promises to the contrary, Australia has not committed significantly to global resettlement efforts (Grewcock, 2017). Not only have the refugees stranded on the now closed detention centres on the Pacific islands not been resettled in Australia, but they have also been made an example of in order to deter others from attempting to enter. Such a rhetoric of deterrence has led to a broader public perception of asylum seekers arriving by boat as “illegal” or illegitimate, and this constitutes a misperception based on inaccurate information that is characteristic of “information disorder” as will be explained in the subsequent section.

Besides international human rights organizations, some local policy think tanks have also critiqued Australia’s political stasis on the issue of boat arrivals. In a report published by the Centre for Policy Development in August 2011, Menadue et al. note:

On the tenth anniversary of the MV Tampa’s rescue of 438 asylum seekers from their distressed vessel Palapa 1, Australia’s asylum and refugee policy is still sadly characterized by human tragedy, political opportunism, policy failure and great cost. People seeking asylum here have been the subject of an increasingly contentious public and political discussion. A toxic debate has polarized large sections of the Australian community and paralyzed politicians of most persuasions from engaging in constructive dialogue. Misrepresentation is rife (Menadue et al., 2011: p. 3).

In a 2015 piece for *The Guardian*, journalist Ben Doherty wrote about the worsening situation on the Manus Island detention centre. He explained that both of Australia’s major parties have ignored “the festering problem of gross abuses on the island, and chosen instead to use the crisis for their own political gain”. At that time, he used the state of the centre on Manus to conclude that on the asylum seeker issue, politics has overshadowed policy.

In the wake of a ruling by the Papua New Guinea legal system that declared the detention centre set up on Manus Island as illegal, the facilities were closed by the Australian government in 2017. Subsequently, 630 of the asylum seekers were “swapped” in a deal with the US, while the rest were relocated to a hotel in Manus and given the choice of moving to the capital of the country, Port Moresby. Media reports indicated that the stranded asylum seekers were afraid to leave the hotel premises due to local attacks (Baker, 2019). In a scathing overview of Australia’s recent asylum seeker policies published in *The Conversation* in 2019, Holbrook called it “a story of blunders and shame”. She attributed these “shambolic attempts” to successive federal governments rejecting the advice of the public service (Holbrook, 2019). This conclusion implies not only Australia’s defiance of international human rights principles, but deliberate and calculated cruelty from federal governments for domestic political ends.

More than a decade since the Menadue report and despite a new Labor government that was attempting to create certainty for refugees on temporary visas on the Australian mainland in 2022, political stalemate for boat arrivals is no better than it used to be. In February 2020, *The Guardian* reported that while the final 18 men who were detained at the Bomana detention centre in Papua New Guinea had been released, they were found to be in a deplorable condition (Taylor, 2020). The year 2019 ended with the Medevac law being repealed by the federal government. Repealing of this law meant that asylum seekers in offshore detention who are found to be in need of urgent and specialized medical attention can no longer be transferred to the Australian mainland (Martin, 2019). As a consequence of a deal between former US President Obama and ex Australian Prime Minister Turnbull, 40 refugees from Manus and Nauru were transferred to the United States in May 2020. This transfer took place despite the nation being in the grips of the COVID-19 pandemic. The case study of a non-Muslim Tamil family, the Biloela family will be examined later in this article to highlight how their treatment classifies as an exception in the broader ecology of information disorder from the state and the national media that perpetuates calculated care towards Muslim asylum seekers and refugees in particular.

In the case of India, as of January 2020, the refugee population was estimated to be over 240,000. Of this, Tibetan refugees form the largest population, followed by Sri Lanka, Myanmar, Afghanistan, and Pakistan (Irudaya Rajan, 2022). The treatment of refugees of different religious backgrounds in India must be understood in relation to the complex fabric of postcolonial nation-building after the British left the subcontinent. As Samaddar (2019) has argued, the anxieties and insecurities of the postcolonial Indian nation are manifest in the lack of clarity about who was considered to be a full citizen, a temporary resident, and those who was to remain stateless.

Despite being one of a handful of countries in the world to have abstained from the 1951 Refugee Convention, India has historically received and supported refugees and asylum seekers from its neighbouring countries. However, the South Asian refugee regime constructs vulnerability and the legitimacy of claims to resettlement based on religio-cultural identity as opposed to the international refugee regime where statelessness is sufficient to lay claim to common humanity and care (Raheja, 2018). Thus, after the Partition of British India in 1947 which resulted in one of the largest mass migrations and refugee crises in modern history, Hindu and Sikh populations displaced by the new national borders were welcomed and resettled as people returning home.

In the absence of a coherent, rights-based policy framework, claims tend to be dealt with on a case-by-case basis with two outdated laws as points of reference: the colonial-era Passport Act of 1920 and the Foreigners Act of 1946. The state is not obliged to make a distinction between asylum-seekers, refugees and other foreigners and holds wide powers of detention and deportation over anyone considered an irregular/undocumented migrant (Irudaya Rajan, 2022).

Muslim refugees from neighbouring countries of Afghanistan, Bangladesh, Myanmar have been sometimes welcomed and allowed to stay as temporary residents but never treated as natives returning home. This calculated management of the political dimensions of hospitality has been comprehensively documented by the scholarship on the massive refugee crisis created by the 1971 War of Independence between Pakistan and East Pakistan, now Bangladesh (Samaddar, 2003). Initially, displaced persons, Hindu or Muslim were welcomed and sheltered in relief camps. However, the discourse quickly shifted from caring hospitality to one of “infiltration by dangerous Muslims”. This discourse continues to dominate media and policy narratives more than four decades later: Bengali-speaking Muslim migrants (even internal migrants) continue to be framed as inherently criminal and disloyal security threats (Ray Chaudhury and Samaddar, 2015). The differential treatment of refugees according to religious identity must be understood against this backdrop of a longer history of prejudice and systemic discrimination that predates independent India and its contemporary manifestation will be explored in the case study of Rohingya refugees explored at length in this article.

### “Information disorder”, migration, and the mediated mobilization of emotions

In the era of “fake news”, various definitions have been in circulation in policy and scholarship about what constitutes false information spreading at a fast rate, now also knows as the phenomenon of “information disorder” that this special issue addresses. According to Wardle surmising these developments in 2018, “Clearly delineating what counts as information disorder is difficult. Legislators struggle with content that might be legal in other contexts—incitement to violence or hate speech—but nevertheless harms individuals, organizations, or even the democratic process. The definition of information disorder is not black and white; it’s fluid” (Wardle, 2018). The seven categories created by Wardle include to encompass where information disorder may take place include satire and parody, false connection, misleading content, false context, imposter content, manipulated content, and fabricated content.

What the above categories emphasise is that information being “inaccurate” is only one aspect of information disorder. What is also overlooked is how these information ecosystems often have ideological underpinnings, such as a long history of misrepresentation of, and racism towards certain communities. According to Vraga and Bode, misinformation includes “misperceptions as cases in which people’s beliefs about factual matters are not supported by clear evidence and expert opinion – a definition that includes both false and unsubstantiated beliefs about the world” (Vraga and Bode, 2020: p. 338). This definition specifies the importance of evidence and expertise in determining what is accurate information versus information disorder. Disinformation, or deliberately created and circulated false information, is a particular concern with regards to discourses about migrants and refugees. This is because the “debate about migration has progressively moved towards the terrain of identity, religion, culture, and social group relations, triggering emotional reactions in target audiences often linked to political projects focused on the protection of national values, traditions, and ways of life” (Komendantova et al., 2023). As mentioned earlier, in the wake of the COVID-19 pandemic and ensuing economic downturns across the globe, disinformation about migrants in host communities has been increasingly amplified (Sengul, 2021). In light of this, we contend that examining disinformation in discourses of migration is particularly important in the current global conjuncture.

For the purposes of this article, Islamophobia is the focus of the disinformation about migrants that we will examine in two nation-states in detail. It is understood here as a structural phenomenon constructed by a set of institutions and policies (as well as discursive and ideological processes) including the state and collective social actors or social movements invested in perpetuating prejudice and violence against Muslims (Massoumi et al., 2017). In a post-9/11 world, state-sanctioned Islamophobia manifests firstly through the ever-expanding counter-terrorism apparatus which includes very large, powerful and unaccountable institutions with close links to multinational technology and security companies (Massoumi et al., 2017). Scholarship on Islamophobia also underscores that both state and non-state actors constitute intricate transnational circuits and modalities through which Islamophobia manifests as well as spreads globally (see, for example, Ganesh et al., 2024). Around the world, the othering of refugees – men, women and children—have become central to state efforts to restrict and weaken the transnational imaginaries encouraged by humanitarianism in their bid to perform the political dimensions of hospitality while maintaining legitimacy and territorial control (for example, Williams, 2015; Raheja, 2018).

This article focuses on disinformation about Muslim migrants and refugees in two nation-states that are seldom considered together. We argue that this is a worthwhile and indeed timely comparison for a number of reasons. Both India and Australia cultivate an international image of themselves as plural, democratic nations that do their share to resettle “genuine” refugees while trying to maintain “sovereign borders”. India has been globally applauded for decades as the country that has sheltered Tibetan refugees (Bentz, 2012) and the Modi regime has worked hard to project itself as “neighbourhood first responder” by sending humanitarian assistance to neighbouring countries afflicted with either natural disaster or security conflicts (Chakradeo, 2020). In Australia, the narrative of “10 pound Poms” – migrants from postwar Britain remains central to nationalist discourse about desirable migrants who were brought in for the project of nation-building.

However, as we show, both nations also share a history of anti-Muslim sentiment which can be traced back to British colonial legacies of Islamophobia. Over the last four decades and despite policy and public commitments to humanitarianism (if not human rights), national refugee regimes in both nations have constructed Muslim refugee and asylum seekers as undesirable and undeserving of care, hospitality and humanity. Juxtapositioning these two contexts has helped us to show the central role played by official and unofficial agents of information disorder that normalize and sustain these uncaring discourses and contingent performances of care and hospitality. This analysis is supported with in-depth literature reviews combined with thematic analysis of mainstream media reporting and political commentary related to the selected refugee case studies. Based on Braun and Clarke’s (2023) recent work on distinguishing positivist thematic analysis from reflexive thematic analysis, we employ the latter as our positioning in relation to both national contexts provides us with particular resources for interpretation. This also means that the themes are generated as interpretive stories rather than pre-determined before the analysis. Reflexive thematic analysis recognises positionality as a strength of interpretation. Therefore, the researchers’ location in both countries assists with making meaning rather than being seen as a hindrance to discerning meaning from the data.

## “Cruel care” for Muslim refugees in India and information disorder

The origins of anti-Muslim discrimination and Islamophobia in India can be traced back to the period of colonialism, marked by the British rulers’ adoption of a divisive strategy famously known as “divide and rule”. Numbering around 150 million today, Muslims constitute the largest minority in India, yet they remain politically and socioeconomically marginalised. Highlighting the lack of development of Muslims, Rahman (2019) argues that although they constitute 14.2% of the population, yet their contribution to the GDP is only about 6%. The majority of Muslims live in poverty and are forced to contend with severe, persistent and violent forms of prejudice and institutionalized discrimination. Given the above, scholars of Islamophobia in the subcontinent argue that an anti-Muslim orientation is constitutive of the post-Independence Indian state. Although charged with building a secular nation, the Indian state and its institutions have worked to consistently discipline Muslim citizens and deny affirmation of their political identity as legitimate historical subjects (Kattiparambil, 2023; Fazal et al., 2024). This work has been enabled and co-constituted with a media ecology where state and non-state actors including legacy news media and Bollywood perpetuate a narrative of Muslims as threats to the Hindu majority (Kumar, 2013).

The main themes in this longstanding disinformation discourse include Muslims as medieval invaders who destroyed and ultimately divided (through Partition) an ancient and great Hindu nation; Muslims as a current threat to the Independent nation of India—untrustworthy, disloyal and working to undermine India in any number of ways including killing cows, engaged in “population jihad” (having multiple children in polygamous marriages) and “love jihad” (by luring Hindu women into marriage and forced conversion) with the final intention of eventually becoming the majority population in India (see also Banaji and Bhat, 2019; Amarasingam et al., 2022). These homegrown narratives which existed long before 9/11 have aligned effortlessly with the resurgence of Islamophobia in the West following terrorist attacks in North America and Europe.

The treatment of refugees from different faith backgrounds has been deeply impacted by the Islamophobic narratives described above. These are the deeply rooted, distorted and dehumanizing narratives about Muslims – citizens and migrants—which have shaped constructions of and responses to Rohingya Muslim refugees in India since their arrival in 2012. These affect-laden misrepresentations have been amplified by the digital misinformation, disinformation, and malinformation which characterize the administration of two-term Prime Minister Narendra Modi and the Bharatiya Janata Party (BJP), the official political party of a 100-year-old Hindu nationalist movement (Chopra, 2019).

Under the current Hindu nationalist dispensation, the distinction between Hindu and Muslim migrants is particularly stark, with the former portrayed as deserving refugees, while the latter are rejected as illegal migrants, criminals and Islamic terrorist threats. This is made clear in the 2014 BJP election manifesto under the sub-heading, “Foreign relations, nation first, universal brotherhood”: “India shall remain a natural home for persecuted Hindus and they shall be welcome to seek refuge here”. Two years after winning the national elections, the Modi administration introduced the Citizenship (Amendment) Bill (CAA) to Parliament in July 2016. The aim of this legislation was to fast-track citizenship for migrants and refugees of all faiths except Islam from the neighbouring Muslim-majority countries of Afghanistan, Bangladesh and Pakistan (not Sri Lanka), that is, Hindus, Jains, Sikhs, Parsis, and Christians. In the north-eastern state of Assam, the BJP administration went several steps further to pilot a National Register of Citizens (NRC) and set up detention camps for those who could not provide the required documentation to prove their Indian citizenship. The vast majority of people interned in these camps are Bengali-speaking Muslims (Amnesty International, 2019). The creation and maintenance of an environment of information disorder has played a vital role in supplanting policy and practice towards the Rohingya which can be generously described as contingent care (Williams, 2015) with unapologetically cruel treatment. In this media environment, a lack of care for stateless Muslims can coexist with increased obligations in terms of governance ideals, processes and authorities to care for and resettle persecuted Hindus from Pakistan and Afghanistan (Raheja, 2018). As we show in the next section, this information disorder has been created and maintained by both official and unofficial agents of disinformation that inculcates Islamophobia (Massoumi et al., 2017; Wardle and Derakhshan, 2018).

## Case study: Rohingyas as the subject of state-sponsored disinformation

Often referred to as the world’s most persecuted minority, the Rohingya have suffered discrimination and displacement since the 8th century when the descendants of Arab and Persian traders populated the Arakan, now Rakhine region of present-day Myanmar. As in India, British colonisers further exacerbated existing hostilities in a region marked by ethnic conflict, pitting the Buddhist Burman majority against local Muslims. Muslim Rohingya have been fleeing their home in Rakhine since the 1960s and 70s when the state excluded them from among the 135 ethnic groups recognised for citizenship and political rights (Nair, 2022). Prior to this, the Rohingya had served as representatives in the Burmese parliament and as parliamentary secretaries, ministers, and in other high-ranking government jobs (Bhat, 2022). They entered India in three major waves in 2005, 2012 and in 2016/2017 through different routes via the West Bengal border or further east through the borders of the states of Meghalaya and Mizoram (Irudaya Rajan, 2022).

Current population estimates of the Rohingya in India in 2018–2019 varied between 20,000 and 40,000 according to UNHCR and the Indian government, respectively (Nair, 2022). However, there is general agreement that exact numbers are difficult to estimate given that this community has been compelled to live an undocumented existence in the shadows. Two thirds are estimated to reside in camps/slums in the northern most state of Jammu and Kashmir followed by the southern city of Hyderabad and the region surrounding the national capital city of New Delhi. Smaller numbers are known to live in the Indian states of Rajasthan, Tamil Nadu, and West Bengal. Wherever they live in India, the Rohingya live in conditions of extreme poverty, deprivation and violence. Men, women and children, who have survived rape, torture, and trafficking are compelled to live in inhumane conditions in temporary accommodations devoid of electricity, water, sanitation and safe access – vulnerable to fire, flood and water-borne diseases. There is little access to health facilities, including for pregnant women and children (Nair, 2022).

However, until 2016, the Rohingya had been able to secure refugee cards or Long-Term Visas (LTV) – vital documentation which allowed them to get *Aadhar* identity cards. These digital identity cards are now required to open bank accounts, obtain drivers licenses, and otherwise participate in the cashless, digital economy imposed on the country by the Modi administration (Tiwari and Field, 2020). Subjecting the undocumented Rohingya to even higher degrees of precarity and vulnerability was not enough for the Modi administration which has been in lockstep with Buddhist nationalists in Myanmar. For instance, when the Myanmar authorities announced that the term, “Rohingya people” was a fabrication, Prime Minister Modi avoided using this word during his 2017 state visit (Amin, 2018). As tens of thousands of Rohingya tried to escape another targeted campaign of ethnic cleansing in their home region of Rakhine, the Indian government denied entry to new refugees. Indian security forces mobilized along the border with Bangladesh and Myanmar and began to arrest and return people to a region where Burmese military had laid landmines. Last but not the least, the Rohingya living in India were subject to rising levels of harassment and violence by police and Hindutva activists.

Our documentation and analysis below show that these violent and restrictive actions were necessarily accompanied by a state-led campaign of mediated disinformation which included elected representatives, state-linked legacy national and local news media outlets as well as a range of unofficial agents including organised and informal media linked to the Hindu nationalist movement. Together, they maintained a culture of managed hostility, or “cruel care” towards the Rohingya dominated by affects that encourage distancing and disconnection, e.g., doubt, disgust, fear, and anger rather than empathy and care for the brutalised and dying refugees. In December 2017, S. Jaishankar, the then Foreign Secretary (and current Foreign Minister) also signed a bilateral MoU with Myanmar for the Rakhine State Development Program.

## Analysis theme 1: elected representatives and state-linked media as official agents of information disorder

The Rohingya were publicly labelled as illegal immigrants and threats to national security by leading government figures including Prime Minister Narendra Modi, Home Minister Amit Shah, and Minister of State for Home Affairs Kiren Rijiju. After Myanmar’s army blamed Muslim Rohingya militants for a mass grave of 28 Hindus in Rakhine, Home Minister Kiren Rijiju stated, “India is not a signatory for refugees, we have been soft. We will facilitate their return”. The government then declared their intention to deport all 40,000 Rohingya and submitted an affidavit to the Supreme Court which cited the potential for radicalisation and reiterated that India did not adhere to the principle of non-refoulement (forcible return) mentioned at the outset of this article (Soni and Sharma, 2017). The Supreme Court then became a battlefield between the government and human rights activists around the unconstitutional deportation order. The Supreme Court rejected the government’s basis for deportations in October 2017, stating that the government “must strike a balance between human rights and national security interests” (Human Rights Watch, 2018: p. 6). However, by October 2018, the government had ordered states to collect biometric data for all Rohingya which would be used to initiate actions for returns through diplomatic channels with Myanmar.

During this same period, pro-Modi English-language TV news channels, namely, Republic and Times Now (and other Hindi news television) helped to disseminate anti-Rohingya propaganda with hashtags designed to provoke fear and rejection. These included hashtags such as #RohingyaTerrorExposed and #SendRohingyasBack (Mohanty, 2020). One television news anchor led the pack—RepublicNow CEO Arnab Goswami, who has built his career on amplifying the exclusionary and hateful narratives of Hindu nationalism. Goswami used his prime time television program, “Debate Hour” to criticise media and policymakers who expressed concern for the plight of Rohingyas in India and beyond. As always, he argued that India had enough problems of its own and did not need any more (Muslim) appeasement. Meanwhile the screen repeatedly flashed a barrage of graphics and provocative statements such as, “Rohingya politics over national security?” (Mohanty, 2020).

## Analysis theme 2: organised unofficial agents of information disorder

Islamophobic statements by government officials were accompanied by anti-Muslim disinformation and malinformation produced by organised social media units and networks linked to the Hindu nationalist movement. Hindu nationalist or Hindutva social media platforms amplified statements by prominent BJP and RSS leaders. These included Braj Bihari Kumar, founding member of Astha Bharati (RSS front) and Chair of the Indian Council for Social Science Research (ICSSR) from 2017 to 2019. Kumar stated that the nation should not tolerate Rohingyas (Pathak, 2017). Similarly, another prominent Hindutva activist and head of the Chamber of Commerce and Industry in the state of Jammu and Kashmir, Rakesh Gupta stated in a press conference that Muslim Rohingyas and Bangladeshis were drug traffickers and should be identified and killed (Mohan, 2018: p. 7). In addition, fake news was produced and propagated through various movement media including regime-friendly television and newspapers.

## Analysis theme 3: unorganised unofficial agents of information disorder

The scale and viciousness of the messaging produced by all of the above agents appear almost moderate compared to the disinformation and misinformation campaigns that played out on social media platforms such as Twitter, Facebook, WhatsApp, and Telegram. As previously discussed, these digital platforms have enabled relentless circulation of anti-Muslim propaganda in the form of memes, and fake or doctored videos designed to escalate fear and hostility towards all Muslims in India. A significant part of this relentless messaging takes the form of “evidence” of persecution of Hindus (and occasionally Sikhs) in Muslim-majority neighbouring countries.

In 2017, a flood of fabricated stories using photographs from unrelated sources flooded the Internet to spread the narrative portraying Rohingya Muslims as terrorists and killers. The abuse of girl children by Rohingya featured prominently in these fake stories. For instance, the Twitter handle of Advocate Prashant P. Umrao (@ippatel), a vocal online supporter of Hindutva, with a following of 22.9 k Twitter followers, posted an image of a pregnant Rohingya refugee girl at a UN clinic. The affective elements of this kind of disinformation which uses images of children cannot be minimized. As Banaji and Bhat (2019) have shown in their landmark study of Hindutva-inclined Whatsapp users, the effect on most viewers is an immediate and visceral provoking of affective states of shock, awe, disgust and even perverse fascination that can significantly disrupt daily flows of life. These stories continued to “trend”, accompanied by anti-Muslim bigotry even after respected investigative journalist website BoomLive correctly identified and sourced the image as that of a sick child suffering from liver disease in Brazil (Rebelo, 2017).

At the same time, these information disorder ecologies ignored or downplayed the death of a 40-day old Rohingya baby. In July 2023, newborn baby Habiba died after inhaling tear gas fired by the police during clashes between detained refugees and the detention centre staff in Kathua district of Jammu. Government authorities were emboldened to claim that the baby had been unwell since her birth even as relatives of the family told a handful of interested reporters that Habiba had been denied medical treatment (Maqbool, 2023). Social media videos that emerged later from the detention centre showed that the police assaulted pregnant women, disabled persons, and sick and elderly persons. However, these verified images and information of extreme suffering failed to evoke any form of care or concern for the Rohingya or their children.

## Tampa, children overboard, and the origins of state-initiated information disorder about refugees in Australia

Recent scholarly commentary and empirical research on the asylum seeker issue in Australia suggests that the origins of current policy and subsequent state-sponsored disinformation on refugees lay in the immediate aftermath of 9/11 when the Tampa and “Children Overboard” crisis took place in Australian waters. According to Patil and McLaren, the above incidents almost co-occurring led to heightened Islamophobia that also characterized how later boat arrivals were handled:

Asylum seekers arriving to Australia by boat increased from 133 in 2008 to 4940 in 2010… Of concern to media were the rising numbers of asylum seekers from places such as Afghanistan, Sri Lanka and Iran… Irrespective that the people from these countries are of different ethnicities, and various religions, and seeking asylum for different reasons, the Australian media tended to uniformly characterize them during these times as Muslim asylum seekers… Media concomitantly held the former Rudd government’s offshore immigration processing strategies as responsible for perceived rising numbers of Muslims migrating to Australia (Patil and McLaren, 2019).

It is also worth mentioning in reference to the Rudd government that although it was elected in 2007 on the platform of a more humane refugee policy agenda, the characteristics of the right-wing media made this impossible (eventually leading to a policy backflip and leadership stoushes in the party that lasted the whole term). In the Australian context, Peterie used discourse analysis of press statements from political leaders to establish that since 2001, governments have constructed varying objects of compassion in the asylum seeker debate, and rendered maritime arrivals as either unworthy or dependent (Peterie, 2017).

In other words, casting all refugees arriving by boat as “Muslim” and therefore undesirable became the go-to disinformation strategy of such media, and especially the publications owned by Rupert Murdoch. They contributed to an ecology of “information disorder” and related anxiety and fear on the asylum seeker issue that has been difficult to dislodge ever since.

The broader context of the racialisation of Muslims in Australia is due to key factors such as Australia being a settler colonial state and the “othering” of Muslims which began long before 9/11 and the federation of British colonies into the Australian nation-state. Poynting and Briskman (2017: p. 138) highlight the role of the settler state in circulating and promoting “Islamophobic commonsense” information about Muslims with little factual basis through both its “coercive apparatus (military, police, courts) as well as its consent-constructive apparatus of hegemony (notably the media)”.

In his book chapter commenting on the characterisation of Muslim refugees in particular, Fethi Mansouri highlights the shifts in these discursive constructions:

The Howard-era rhetoric around the “illegality” of people arriving by boat, as well as the criminality surrounding people smugglers, were amplifications of earlier discourses, which, in turn, have helped determine contemporary public suspicion of non-white newcomers to Australia, in particular adherents to the Islamic faith … Australia, as a settler-colonial state, is founded on the dispossession of Indigenous people from their lands, and the myths of nationhood which actively obfuscate this—such as the notion of Australia being terra nullius (the land of nobody)—enabled the emergence of a white nationalism. This nationalism led to the White Australia policy and still underlies Australian public discourse, clear in the ongoing racialisation of non-white Muslim and Middle Eastern asylum seekers (Mansouri, 2023).

Within the larger context described above, the global and local specificities of 9/11 and the “Children Overboard’ saga contributed to further demonisation of Muslim refugees in the Australian context. According to Caluya, 2001 marked a turning point of sorts when “the figure of the refugee changed in the Australian public eye from being victims of rape and sexual assault in war to being potential perpetrators lurking among ‘genuine’ refugees, preying on ‘our’ goodwill” (Caluya, 2019). In the social media space, this continues to the present day as reporting on international “criminal” activities attributed to Muslim refugee men appears in “Australian anti-refugee and anti-immigration lobby groups in online forums and shared by popular conservative leaders in social media” (Caluya, 2019).

In the era of disinformation and fake news, there is heightened fear of not just boat arrivals, but also the sources of information that people rely on to understand domestic and international news. Haw’s work on Australian citizens’ perceptions of asylum seekers in this new era shows that some use the “fake news” label to discredit all news media that contradict their ideological position, while those sympathetic to refugees and asylum seekers usually view media agencies as either deliberately or inadvertently misleading the public about people seeking asylum (Haw, 2021: p. 780). In the wake of new global crises likely to produce refugees, such as in Ukraine and Gaza, further research could help us understand Australian media consumers’ sources of news and trust in the same, and how it mobilizes fear or conditional forms of care.

## Information disorder and Muslim refugees in Australia

Beyond the seminal events that shaped the asylum seeker policy agenda described above, other occurrences in the past decade illustrate how the “information disorder” has both continued and become more complex. When the Australian federal government announced in 2017 that the Manus Island detention centre would close due to the PNG court ruling, it was widely noted that detainee protests around this time were barely covered in the national media. For instance, international publication *Al Jazeera* reported:

The story is not, by any measure, leading every news bulletin. Despite restrictions on reporting from the centre, there has, however, been more interest in the plight of these men this week than there has been in months.

Still, large sections of the public are hostile to the cases of these men; many others are just apathetic (Code, 2017).

An episode of ABC’s *Media Watch*, a show on the nation’s public broadcaster, also covered the Australian media’s relative lack of reporting on an issue that was being extensively noticed by the international press. The program suggested that this was due to the Department of Home Affairs not issuing visas to Australian journalists to enter PNG despite alleging that this decision was out of their hands (ABC Media Watch, 2017). In other words, both the public’s long-standing apathy regarding Manus, and the federal government’s disinformation agenda contributed to the informational disorder ecology locally.

It has also now become clear that the right-wing news media in Australia and social media echoed each other in terms of anti-migration and Islamophobic discourse during particular asylum seeker-focused coverage. Gallagher notes that “the discourses of News Corp Australia are largely the same as the Alternative Influence Network (AIN) on YouTube – a loosely connected group of reactionary right-wing influencers” (Gallagher, 2019). He explicates that this takes place discursively as both kinds of media discriminate against a number of minority communities by centring a white, western identity as the norm (Gallagher, 2019). Finally, he describes New Corp’s activities as “ideological misinformation”, and this is a crucial moniker for future research on misinformation such that its ideological underpinnings are not overlooked in the search for “objective” facts.

Together, they maintain representations of Muslim refugees that overwhelmingly evoke negative affective responses—of fear, disgust, anger—which distance the recipient from the subject of the media. The media representation of refugees in Australia has received attention since the early 2000s, especially with regards to how they are depicted in news discourses (Pickering, 2001; Gale, 2004; Klocker and Dunn, 2003). Several scholars have identified an overarching Judeo-Christian settler ethos in these representations accompanied by distancing communicative devices of spectacle which make the Australian government appear less hostile and less responsible (Szörényi, 2006; Stratton, 2009). This can be seen most clearly in conservative media outlets in Australia where refugees and asylum seekers are routinely and casually dehumanized via tactics such as visual framing, not showing individual asylum seekers, and associating them with threats to border security (Bleiker et al., 2013).

Further work on the detainee protests at Manus in 2017 has uncovered that despite the persistence of state cruelty and disinformation, refugees themselves resisted via digital means. Sharples notes that this is reflective of a broader trend whereby “asylum seeker use of social media in this modern era of offshore detention is both unique and innovative” (Sharples, 2021: p. 5). Despite the above-mentioned government restrictions on media access to the detention centre, asylum seekers’ own social media accounts have been a crucial source of information (Sharples, 2021: p. 5). This is especially true of the Twitter posts and subsequent commentary for *The Guardian* provided by former Kurdish refugee and writer, Behrouz Boochani who was also held at Manus. Not only did this lead to eye-witness accounts at the time of closure of the detention centre which would otherwise not have been possible, but it also enabled the wider Australian public to view a “refugee” like Boochani through the lens of an informed, multi-dimensional person rather than simply an “illegal” or a “queue jumper” (Sharples, 2021: p. 5), thereby briefly disrupting the environment of information disorder.

## Case study: the Biloela family and “calculated care”

Also disruptive of prevalent logics and feelings was the case of the “Biloela family” that broke the norm of state treatment of, and public feelings for refugees in the Australian setting in recent years. This case study has been chosen for analysis here as it was one of the few cases of “exception” where state cruelty turned into state “calculated care” towards a refugee family due to a very effective grassroots campaign (initiated by “ordinary people” living in a regional town rather than a left-wing or refugee advocacy organisation) and its affective mobilization of the Australian public. Even then, the exception was likely made because a non-Muslim family perceived as non-threatening and hardworking, and contributing to the economy of a regional town in Queensland was involved.

While this case study may not appear to exemplify disinformation about refugees in the Australian context at first glance, it is important to remember that the family was treated as exceptional precisely because of the norm that is the criminalisation of boat arrivals since Tampa (itself based on state-sponsored disinformation as outlined in the sections above). This means that public grassroots campaigns such as the one mounted for the Biloela family in the Australian setting are always attempting to speak back to discursive disinformation about refugees, and often doing so without adopting radical strategies such as calling for an overhaul of all contemporary policies of state cruelty. These campaigns have centred on the treatment meted out to refugee children in detention to evoke broader public sympathy (Khorana, 2022), and this can also be seen in the case of the Tamil family with two Australian-born daughters.

## Analysis theme 1: “normalizing” a non-Muslim asylum seeking family

This second case study examined in the article will entail a reflexive thematic analysis of the #HometoBilo campaign—a grassroots and digital campaign in Australia to bring a Tamil refugee family back to their Queensland “hometown” of Biloela and beat the fate meted to most boat arrivals in the country. Although this example is focused on a non-Muslim family, it has been chosen deliberately to demonstrate how (a) such a popular campaign could have only been mounted for a family without any connection to Islam and with two young daughters born in regional Australia; (b) even with its popularity, it was subject to disinformation and propagandist media coverage from the nation’s right-wing media.

The (women-identifying) social workers from Biloela responsible for initiating the campaign deliberately used discursive strategies to “normalize” the Tamil family. Speaking of Nades, the father, they painted the picture of a hard worker who wasn’t turned off by manual work; and in the case of the mother, Priya, they conjured up the figure of a non-threatening woman of color who contributed to the community, often through her cultural practices:

His first job here was pushing trolleys at Woolworths. Nothing was below him or beneath him, he didn’t feel he was better than the next person in the street. Aussies in general value that, and particularly country people value that … Priya threw herself into community life. She went to crafting classes and volunteered. She made curries and brought them to staff at the nearby hospital. She would wave as she marched the same circuit around Biloela, every afternoon at the same time, pushing a stroller (Dendle cited in Smee, 2021).

As if to set up a contrast with the refugees allegedly involved in the “Children Overboard” saga, Nades and Priya were also set up as “attentive parents”, thus making the family easy for the Biloela community to admire and accept (Smee, 2021).

The above discursive framings were successful in mobilizing many otherwise apathetic communities and individuals. Among these was Barnaby Joyce, the leader of the Nationals Party (the Australian political party that has the strongest mandate in regional towns). Pleading the Coalition government for a change in stance towards the Biloela family in 2019, he also highlighted their regional contributions: “A family that’s not making the traffic more difficult in Sydney or Melbourne or Brisbane, that are working in a regional area doing jobs that other people may not be willing to do, that are well supported by their local community” (Joyce cited in Hunter, 2019). At the same time, former Prime Minister Scott Morrison was seen by the Home to Bilo campaign as engaging in misinformation by claiming that the family’s matter was before the courts in 2022, and also by stating that visas cannot be granted to them without a finding that the family needs protection (No Author, 2022). In other words, the campaigners were labeling the historical policy of referring to boat arrivals as illegal as a piece of misinformation as this defied the internationally-accepted definition of a refugee as per the UN’s 1951 Refugee Convention.

## Analysis theme 2: care reserved for “deserving refugees”

As the campaign itself gained more influence in the lead up to the 2022 federal election, the family became symbolic of public feelings of care towards (deserving) refugees becoming crystallized in one site rather than the compassion spreading outwards to include all refugees facing uncertainty. Writing for the publication *Eureka Street*, Kampark commented on the changing tides of this show of compassion:

The family have since become an example of instrumental and political convenience. The most obvious policy response would have been to return them to Biloela. But to do so, according to Agriculture Minister David Littleproud, would be to succumb to “public opinion and the mob”… In the political stock exchange, the value of keeping the Murugappan family in hostile conditions away from Biloela was diminishing. Conversely, the value of showing compassion and returning them to the mainland was growing (Kampmark, 2021).

In the reporting on the Biloela family that took place in mainstream, feminized media outlets like “Mamamia”, the lens of compassion was again highlighted over structural issues with the nation’s refugee policies. They particularly questioned the government over the “lack of compassion” meted out to the young children when they were in detention in Melbourne, and again when the younger daughter, Tharnicaa had to be evacuated to Perth from the Christmas Island detention centre as her condition was critical (Jepsen, 2021).

As mentioned above, it also matters to the mobilization of calculated care that the “Home to Bilo” campaign was started by “ordinary” women who used many aspects of the campaign to counter the “stop the boats” messages of several federal governments. Even though no one in the core group of campaigners had media training or public relations experience, they made extensive use of crowdfunding to pay for the family’s legal costs, fund 22 billboards in Coalition electorates in Sydney, Melbourne and Brisbane that featured giant photos of the family, and messages imploring the government to let them return to the small town from which they were taken (Hyland, 2021).

One of these messages was directly addressed then-PM Scott Morrison, who also happens to be the father of two daughters: “Please, Prime Minister, you can bring these girls home to Biloela #hometobilo”. Hyland add that “Home to Bilo” was a phrase repeated by the three core campaigners wherever they went and whenever they gave media interviews such that it soon became a “political earworm” just like the words: “Stop the Boats” (Hyland, 2021). Angela Fredericks, one of the leaders of the campaigning team was convinced that their success is owed to being able to show the faces of the family, which in turn “humanized” boat people for the broader Australian public. It is difficult to ascertain whether disinformation about refugees has been completely overcome as a result of a single campaign, but its success does demonstrate that public feelings can be made to matter in certain political conditions. At the same time, these conditions may never be created for Muslim refugees.

## Concluding notes: colonial connections and the role of disinformation in enabling “calculated care” for refugees

In this article, we have explored the nexus between official agents of disinformation—namely the state, news media institutions and unofficial but also collective actors that produce the information disorder that normalises and legitimises the discriminatory treatment of Muslim migrants and refugees in India and Australia. We have identified and discussed elements of information disorder as well as the phases (that is, the creation, production, and distribution) of information disorder (Wardle and Derakhshan, 2018). In both contexts, we have observed toxic techno cultures and particularly a sustained campaign of disinformation—that is, the deliberate spread of falsities, designed to carry emotional content that triggers strong emotions in those who access it, that has resulted in considerable harm to Muslim refugees.

Given the focus of this special issue, we conclude with three overarching observations and a final recommendation about the generation of mis/disinformation and how disinformation shapes larger emotional/affective climates and cultures with two caveats. First, we reiterate that we are in agreement with media scholars who argue that such research must be situated in time, space and place. Digital and other communication practices always take place within particular socio-political contexts and in turn shape socio-political contexts (Banaji and Bhat, 2019). Relatedly, online and offline violence are deeply interconnected, enable unprecedented forms of harm, and influence each other on local, national and international levels depending on constellations of power (see, e.g., Polak and Trottier, 2020). Our discussion about the spread of disinformation about Muslim migrants and refugees and affective outcomes is therefore situated at the intersection of the technological and the social. What we foreground is not just these intersections, but the deep-rooted histories of misrepresentation of minorities by the state that emphasise the need to re-centre the social in the largely technologically-focused research on information disorder. Relatedly, we do not argue that calculated and contingent performances of care or care-lessness is in any way new. Global refugee policy and practice has always been determined by a racialized hierarchy of deserving and disposable refugees and asylum seekers (Bauman, 2003; Kirkwood, 2017). At the same time, amplification through social media does make these messages more potent and harder to correct in the longer term due to the public feelings they have already mobilized to this point. To counter this, research resources focused on regulation are much needed. Our work is part of an emerging body of research that suggests that underlying narratives of racism and Islamophobia require equal attention and resourcing so that regulatory approaches do not simply end up applying band aids to ongoing and long-standing social issues.

Our first observation relates to the role of the state and state-linked media in the production and distribution of disinformation about Muslim refugees. Our inquiry adds to the scholarship that highlights the structural as well as systematic aspect to the production and distribution of disinformation and misinformation. The Hindu nationalist governing party has a far-reaching and well-established media infrastructure which includes movement media, stake-linked media and state-intimidated media. The Australian state – all the arms of government including laws, courts, policing, educational institutions also play a central role in normalizing Islamophobia along with corporate news media outlets like Murdoch-owned News Corp and white supremacist social media. Thus, our analysis underlines the fact that the state is far from neutral on the issue of disinformation and Islamophobia (Massoumi et al., 2017) and therefore we cannot uncritically appeal to the state to arbitrate on questions of the fair representation of, and care for Muslim refugees.

Our second observation relates to the links between the affects of shame and fear of the other (Salmela and Von Scheve, 2017). During the three-decade time period in which our analysis is situated, both nations have struggled with intense economic crises that elected leaders have visibly struggled to manage. In both contexts, external forces or rather people constructed as dangerous and undesirable outsiders have been blamed for all the problems confronting the nation. What is different now, perhaps, is that political leaders are openly participating in the generation and dissemination of disinformation in order to maintain the disadvantaged status of Muslims. It is for this reason that this article recommends more attention to the role of the state in creating and maintaining information disorder in both Global North and Global South contexts.

In conclusion, the manipulation of emotions through mis-and disinformation about refugees is deeply intertwined with historical dimensions of Hindu ethno-religious nationalism (in India) and Islamophobia (in Judeo-Christian societies) as well as the added contemporary influence of algorithmic media ecosystems. Resentment and the manipulation of affect and psychological mechanisms that we have seen as central to the rise of authoritarian populist and ethno-religious regimes can also be seen in the forked tongue through which nation-states talk about Muslim refugees today. This symbiotic relationship between state and media disinformation – particularly digital disinformation – and existing suspicions, fears and feelings of hatred towards Muslims in both societies work together to create and maintain the emotional distancing which permits some refugees to be treated as “less than” others. Therefore, we are hopeful that this research makes a small contribution to the gap in the scholarship about situated country/context-specific socio-political analyses about mis-and disinformation and affect.
